# Sex Pheromone Aerosol Emitters for *Lobesia botrana* Mating Disruption in Italian Vineyards

**DOI:** 10.3390/insects14030270

**Published:** 2023-03-08

**Authors:** Giovanni Benelli, Renato Ricciardi, Francesca Cosci, Andrea Iodice, Edith Ladurner, Francesco Savino, Andrea Lucchi

**Affiliations:** 1 Department of Agriculture, Food and Environment, University of Pisa, Via del Borghetto 80, 56124 Pisa, PI, Italy; 2 CBC (Europe) Srl, BIOGARD Division, Via Zanica, 25, 20050 Grassobbio, BG, Italy

**Keywords:** behavior-based control, European grapevine moth, insect pest, Integrated Pest Management, semiochemical, Tortricidae

## Abstract

**Simple Summary:**

Within the existing pheromone-based strategies for *Lobesia botrana* monitoring and management, mating disruption appears to be the most studied and deployed in wine-growing contexts worldwide. The effectiveness of this strategy led to many efforts to improve it, relying upon new available technologies. In this study, we assessed the efficacy of a novel active pheromone emitter (product code: Isonet^®^ L MISTERX843) for *L. botrana* mating disruption, testing it at three different densities (i.e., 2, 3 and 4 units/ha). The aerosol emitter was evaluated in two wine-growing contexts, where it significantly reduced the *L. botrana* infestation when compared to vineyards not managed with mating disruption. The aerosol efficacy performances were comparable to those obtained with passive dispensers (Isonet^®^ L TT) and another active dispenser (Checkmate^®^ Puffer^®^ LB) already on the market. Overall, this novel aerosol device ensures the high effectiveness of the mating disruption program. On the other hand, extra care should be given for their deployment in the field, considering many factors such as the orography and shape of the vineyard as well as the dominant winds.

**Abstract:**

Despite the great amount of information on the European Grapevine Moth (EGVM), *Lobesia botrana* (Lepidoptera: Tortricidae), and the effective strategies available for its management, this moth remains the main key pest damaging grapevines in the Mediterranean and Central Europe wine-growing areas. Synthesizing and manipulating its sex pheromone components fostered the development of new dispensers to boost the effectiveness and sustainability of mating disruption (MD) programs. Recent MD research has highlighted that the effectiveness of aerosol emitters is comparable to that of passive dispensers when applied in large, uniform sites such as Spanish vineyards. However, aerosol emitters that are equally effective in geographical areas characterized by small-sized vineyards, typical of many Italian regions, have not received enough research attention. To face this challenge, herein the experimental aerosol emitter (product code: Isonet^®^ L MISTERX843) was tested at three different application rates (i.e., 2, 3 and 4 units/ha) in three study sites, two in Tuscany (Central Italy in 2017 and 2018) and one in Emilia-Romagna (Northern Italy in 2017), respectively, for a total of five trials. To assess the efficacy of this novel MD aerosol emitter, three different application densities were compared with an untreated control and two grower’s standards. The latter were represented by passive (Isonet^®^ L TT) and active (Checkmate^®^ Puffer^®^ LB) release dispensers, already on the market for EGVM MD and applied at, respectively, 200–300 and 2.5–4 units/ha. MD carried out with Isonet^®^ L MISTERX843 led to zero catches of males in the pheromone traps. They also allowed for a significant reduction in the number of infested flower clusters and bunches, as well as in the number of nests per flowers cluster/bunch, if compared to the untreated control. As a general trend, MD effectiveness was fully comparable, or even better, if compared to the grower’s standard. In conclusion, our research pointed out that the Isonet^®^ L MISTERX843 can allow for effective EGVM management in small-sized Italian vineyards. Lastly, our economic evaluation showed that the MD whole cost per hectare using active or passive release devices was comparable.

## 1. Introduction

The European grapevine moth (EGVM) *Lobesia botrana* is still one of the most feared grapevine pests in Central European and Mediterranean wine-growing areas [1,2,3,4], as well as in Chile and Argentina [5,6,7]. EGVM also caused severe damages in Californian vineyards, where it had been accidentally introduced and, at present, is considered to be eradicated [6,8,9,10,11]. At Italian latitudes, this moth species can complete three to four generations, becoming extremely dangerous and harmful during the second (G2) and third (G3) generation, feeding on green and ripening bunches, respectively [1]. Over the years, EGVM management has been substantially improved, aiming to limit the use of pesticides [12], to face the development of insecticide resistance [13] and to lower the non-target effects of insecticides on human health and the environment [14,15]. Although several effective strategies are available for EGVM management, this moth is still a fearsome pest in most wine-growing areas, requiring steady monitoring to manage its populations [16].

Baseline knowledge on the chemoecology routing EGVM courting and mating [1,17,18] as well as the technological skills to synthetize and formulate the main components of the pheromone blend leading to its sexual communication [19] allowed the consolidation and ongoing improvement in mating disruption (MD) techniques to manage this key pest. MD dispensers can essentially be divided into two categories based upon the mechanism of pheromone blend release: passive- and active-release dispensers. Passive-release dispensers, made of plastic [20,21,22] or biodegradable materials [23], should be deployed in the field at relatively high densities, i.e., generally 200–500 dispensers/ha, depending on the type, requiring more time and manpower compared to that needed for the installation of the latest generation of active-release emitters (i.e., 2–5 emitters/ha) [24]. Of note, research testing active-release emitters for pheromone blends has outlined that their effectiveness is comparable to passive release emitters, especially when applied in large, uniform sites such as Spanish wine-growing areas [24,25]. Starting from this scenario, advanced emitters that are equally effective in wine-growing contexts characterized by a fragmented and irregular vine area (e.g., small vineyards of few hectares close to each other and with different orography), typical of many Italian regions characterized by high-value vineyards, such as Tuscany (Central Italy), must still be developed. In this scenario, a further question to deal with is “what is the optimal density of aerosol units per hectare?”

In this research, the efficacy of the Isonet^®^ L MISTERX843 (CBC Europe, Div. Biogard, Italy) experimental aerosol emitter for EGVM MD was assessed during two years across three study sites. The experimental design tested three aerosol emitter densities (i.e., 2, 3 and 4 units/ha) in two Tuscan wine-growing areas (Central Italy) as well as in Emilia-Romagna (Northern Italy), comparing their effectiveness with passive (Isonet^®^ L TT) and active (Checkmate^®^ Puffer^®^ LB) dispensers currently marketed. The performance of this novel experimental emitter was evaluated by analyzing and quantifying the damage caused by EGVM in the three generations (G1, G2 and G3) and comparing the results with those of an untreated control and MD growers’ standards.

## 2. Materials and Methods

### 2.1. Aerosol Pheromone Dispensers and Experimental Sites

The aerosol dispenser Isonet^®^ L MISTERX843 is composed of a pressurized unit containing the main component of the EGVM synthetic pheromone (*E*,*Z*)-7,9-dodecadienyl acetate (7–12% *w*/*w*) mixed with isopropyl alcohol (40–50% *w*/*w*) and dimethyl ether (40–50% *w*/*w*), giving a total volume of 180 g; the unit is integrated into an electronic control device, the emitter, used to set and manage the release of the pheromone mixture. The latter was sprayed at regular time intervals over a period according to the flight time of the target moth (i.e., from dusk to midnight [26]) and environmental conditions (mainly temperature and wind). The pheromone release takes place above the minimum threshold temperature of the insects mating flight. The aerosol dispensers were fixed at the top of the row posts with the nozzle pointing towards the row spacing.

Herein, MD tests were performed in 2017 and 2018 on different wine grape varieties in two wine-growing regions. In 2017, trials were carried out in three study sites: the first one was a vineyard of the Cabernet Sauvignon variety located in Castiglione della Pescaia (Tuscany, Central Italy), the second one was a vineyard of Cabernet Franc in Castagneto Carducci (Tuscany, Central Italy) and the last one was a vineyard of the Trebbiano variety in Ravenna (Emilia-Romagna, Northern Italy) (Table 1). Although Cabernet Franc and Trebbiano are more vigorous varieties than Cabernet Sauvignon, considered to be of medium vigor, they have been managed through green pruning to achieve a good vegetative–productive balance comparable to Cabernet Sauvignon. In 2018, the MD trials were performed only in Castiglione della Pescaia and Castagneto Carducci in the same vineyards studied during 2017 (Table 1). 

In all study sites, including the vineyards used as the growers’ standard and untreated control, MD was performed in the two years before 2017 using passive-release dispensers. Further details about the study sites are provided in Table 2. 

Isonet^®^ L MISTERX843 was tested at three application rates in 2017 (i.e., 2, 3 and 4 units/ha), and at two application rates in 2018 (i.e., 3 and 4 units/ha) (Table 3) following the EPPO PP 1/264 guideline that sets 100 m as the minimum distance between plots. The efficacy of this aerosol emitter was compared with MD emitters currently marketed, i.e., Isonet^®^ L TT (ShinEtsu, Chiyoda, Tokyo, Japan) and Checkmate^®^ Puffer^®^ LB (SUTERRA Europe, Valencia, Spain). Untreated plots were also examined, where possible (Table 3).

### 2.2. Experimental Design

To properly assess MD effectiveness, it is important to work on large and uniform surfaces [22,23,24]. Accordingly, the MD treatments (Isonet^®^ L MISTERX843 at 2, 3 and 4 units/ha, respectively, and the grower’s standard) (Table 3) were developed on areas of about 3 ha each with a relatively regular shape, while the untreated control plots were about 1 ha each. The standard, depending on the context, involved different emitters for EGVM MD (i.e., Checkmate^®^ Puffer^®^ LB and Isonet^®^ L TT in Emilia-Romagna and Tuscany, respectively), already on the market.

Following the protocol adopted in recent studies [22,23], the experimental vineyards were divided into 10 sampling sub-plots, at least 200 m^2^ large, to collect and check a minimum of 100 inflorescences/bunches per sub-plot for a total of 1000 inflorescences/bunches per plot at the end of the first and second generation (henceforth G1 and G2), and a total of 500 bunches (Tuscany) or 1000 bunches (Emilia-Romagna) per plot at harvest (henceforth G3). To make a realistic efficacy assessment, all study plots within each study site had a comparable pest history (Table 1). All pheromone dispensers were deployed in the second half of March, before the beginning of the first EGVM flight.

### 2.3. Flight Monitoring of Lobesia botrana Males

The flights of EGVM males were monitored using Biogard Delta Traps (BDT) baited with pheromone lures containing the main component of the EGVM synthetic sex pheromone, (*E*,*Z*)-7,9-dodecadienyl acetate (CBC Biogard, Grassobbio, Italy). Each trap was checked weekly; the pheromone lures were replaced every four weeks.

### 2.4. Mating Disruption Efficacy Assessment

The efficacy assessment of Isonet^®^ L MISTERX843 against EGVM was carried out through sampling in specific phenological phases of the grapevine and considering the EGVM life cycle. The first sampling was performed on 100 inflorescences per sub-plot in the phenological phase of full flowering (BBCH scale 65) on the anthophagous generation (G1), by determining the number of infested inflorescences and the number of nests per inflorescence. The second and third sampling were performed on the two EGVM carpophagous generations (i.e., G2 and G3) by checking respectively 100 bunches per sub-plot at the phenological stages of berries beginning to touch–majority of berries touching (BBCH scale 75–77) and 50 bunches per sub-plot at berries ripe for harvest (BBCH scale 89), respectively.

### 2.5. Statistical Analysis

All the collected data, i.e., infested inflorescences/bunches (%), number of nests per inflorescence/bunch and the number of weekly caught males per trap, showed that they were neither normally distributed (Shapiro–Wilk test, *p* < 0.05) nor homoscedastic (Levene’s test, *p* < 0.01). Data transformation did not allow to normalize the distribution or homogenize the variance. Therefore, for each trial, differences between treatments (i.e., Isonet^®^ L MISTERX843 tested at 2, 3 and 4 units/ha, the grower’s standard MD products, respectively, Checkmate^®^ Puffer^®^ LB and Isonet^®^ L TT, and untreated control), in EGVM-infested inflorescences/bunches, the number of nests per inflorescence/bunch and weekly male catches were analyzed using the Kruskal–Wallis test followed by Steel–Dwass multiple comparison; *p* = 0.05 was the threshold to assess significant differences. Statistical analyses were run using JMP^®^ PRO 16 (SAS Institute, Cary, NC, USA, 1989–2021).

## 3. Results

### 3.1. Lobesia botrana Male Catches

**Figure 1:**
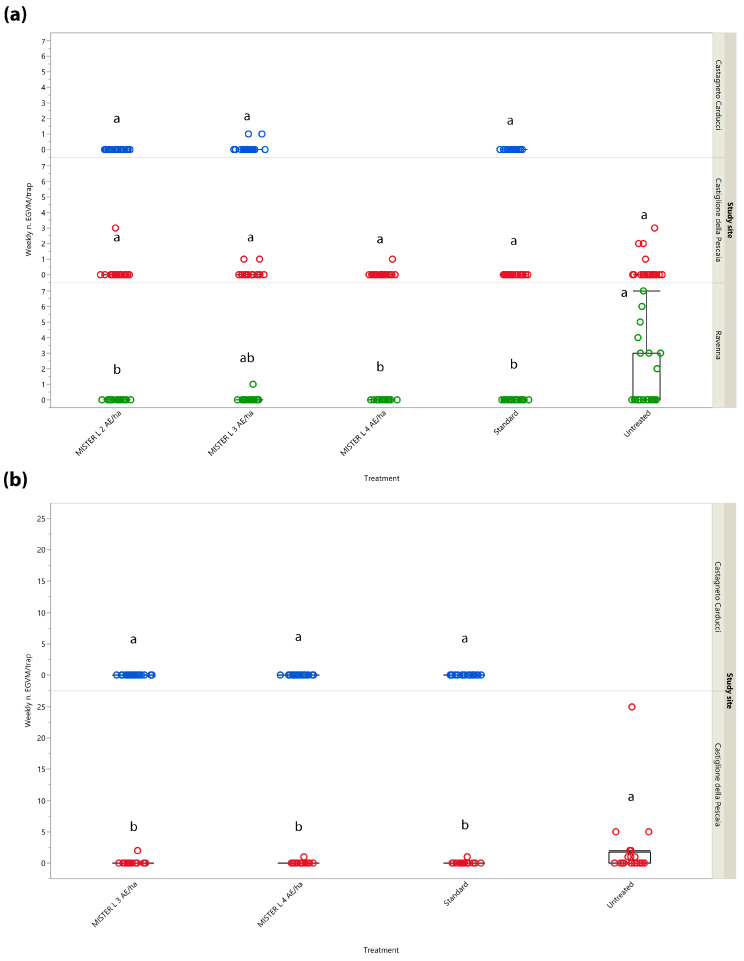
Box plots show weekly *Lobesia botrana* male catches in mating disruption treatments and standard and untreated plots in Emilia-Romagna (Northern Italy, in green) and Tuscany (Central Italy, in blue and red) in 2017 (**a**) and in Tuscany in 2018 (**b**). AE = aerosol emitters. Box plots indicate the median (solid line) within each box and the range of dispersion (lower and upper quartiles and outliers) of the male catches. Different letters above box plots indicate significant differences between treatments (Kruskal–Wallis test followed by Steel–Dwass test, *p* < 0.05).

Results concerning weekly trap catches in the different study sites and years are summarized in Figure 1. Regarding the trials conducted in Central Italy during 2017, no significant differences in the catches were noted among the different treatments tested in the two Tuscan sites (Castagneto Carducci: *χ*^2^ = 4.055, *d.f.* = 2, *p* = 0.132; Castiglione della Pescaia: *χ*^2^ = 6.240, *d.f.* = 4, *p* = 0.182). Concerning Northern Italy, significant differences between treatments emerged for the Ravenna site (*χ*^2^ = 29.806, *d.f.* = 4, *p* < 0.0001). More males were caught in the untreated control than in the MD treatments consisting of Isonet^®^ L MISTERX843 at 2 (*Z* = 3.054, *p* = 0.019) and 4 units/ha (*Z* = 3.054, *p* = 0.019), and of Checkmate^®^ Puffer^®^ LB at 3 units/ha (*Z* = 3.054, *p* = 0.019).

In 2018, male catches substantially differed between the two Tuscan study sites. While no significant differences were noted among the treatments tested in Castagneto Carducci (*χ*^2^ = 0, *d.f.* = 2, *p* = 1), all being equal to zero, significant differences were recorded in Castiglione della Pescaia (*χ*^2^ = 15.938, *d.f.* = 3, *p* = 0.001). More males were caught in the untreated control over all MD treatments (untreated control vs. Isonet^®^ L TT: *Z* = 2.666, *p* = 0.038; untreated control vs. Isonet^®^ L MISTERX843 at 3 units/ha: *Z* = 2.573, *p* = 0.049; untreated control vs. Isonet^®^ L MISTERX843 at 4 units/ha: *Z* = 2.666, *p* = 0.038).

### 3.2. Impact of Mating Disruption on Lobesia botrana Infestation

#### 3.2.1. Year 2017: Infested Flower Clusters and Bunches

**Figure 2:**
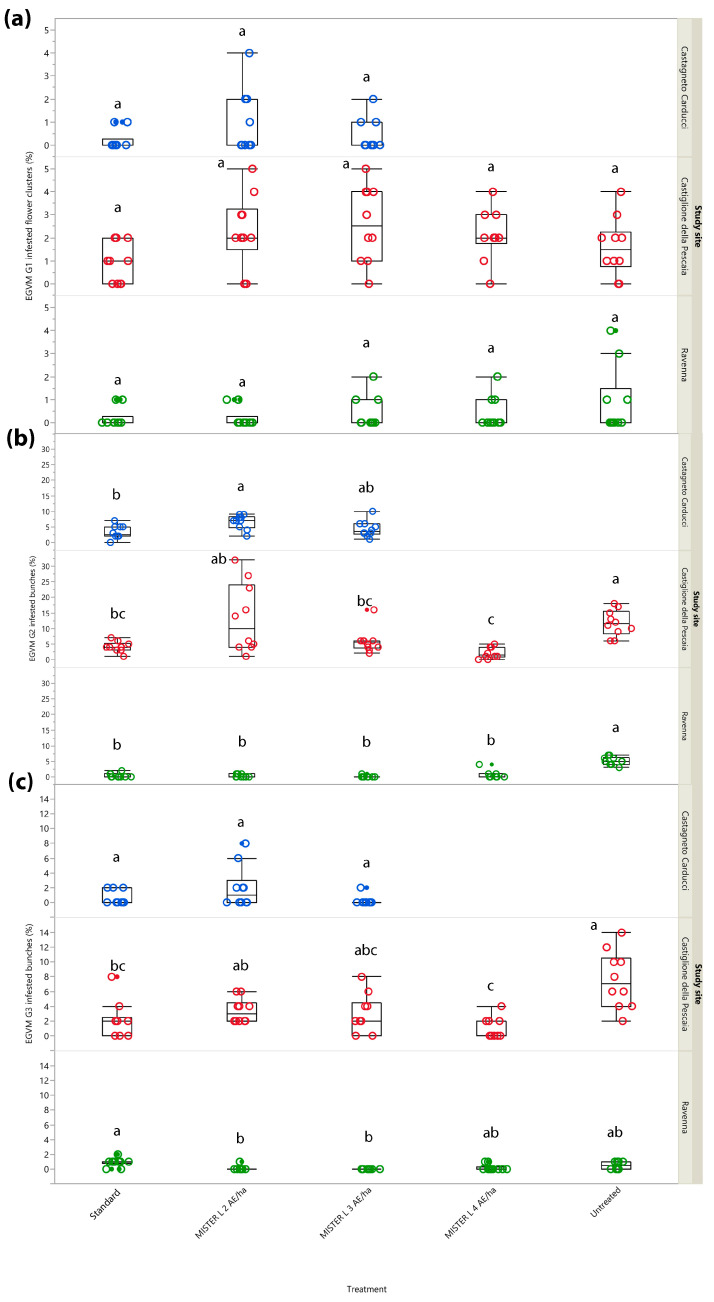
Impact of mating disruption using Isonet^®^ L MISTERX843 on the percentage of flower clusters (G1) or bunches (G2 and G3) infested by *Lobesia botrana* (EGVM) in Tuscany (Central Italy, in blue and red) and Emilia-Romagna (Northern Italy, in green) during 2017. Box plots indicate the median (solid line) within each box and the range of dispersion (lower and upper quartiles and outliers) of the infestation parameter. (**a**) G1: first generation; (**b**) G2: second generation; (**c**) G3: third generation (harvest). Within each generation and study site, different letters above box plots indicate significant differences between treatments (Kruskal–Wallis test followed by Steel–Dwass test, *p* < 0.05).

Both in Northern and Central Italy, G1 samplings revealed a lack of significant differences in the percentage of infested inflorescences between the different treatments (Castagneto Carducci: *χ*^2^ = 1.595, *d.f.* = 2, *p* = 0.450; Castiglione della Pescaia: *χ*^2^ = 9.087, *d.f.* = 4, *p* = 0.059; Ravenna: *χ*^2^ = 2.012, *d.f.* = 4, *p* = 0.733) likely because pest pressure was extremely low in all study sites. Indeed, infested flower clusters did not exceed 5% in any of the tested treatments, with the untreated control included (Figure 2a). On the other hand, G2 infested bunches (%) showed significant differences between treatments in all study sites. In Castagneto Carducci, significant differences (*χ*^2^ = 8.145, *d.f.* = 2, *p* = 0.017) were found between the treatment testing Isonet^®^ L MISTERX843 at 2 units/ha and the grower’s standard, where Isonet^®^ L TT at 250 dispensers/ha was deployed (*Z* = 2.650, *p* = 0.022) (Figure 2b). Both in Castiglione della Pescaia (*χ*^2^ = 24.569, *d.f.* = 4, *p* < 0.001) and Ravenna (*χ*^2^ = 30.395, *d.f.* = 4, *p* < 0.0001) the percentage of infested bunches was significantly higher in the untreated control than in all the MD treatments, among which no differences emerged (Figure 2b).

In G3, no significant differences were found among tested and reference MD dispensers (untreated control not included in the trial design) in Castagneto Carducci (*χ*^2^ = 4.253, *d.f.* = 2, *p* = 0.119), while in Castiglione della Pescaia significant differences were found (*χ*^2^ = 21.192, *d.f.* = 4, *p* < 0.001); a lower percentage of infested bunches was recorded in the plots where Isonet^®^ L MISTERX843 at 4 units/ha (*Z* = 3.546, *p* = 0.004) and Isonet^®^ L TT (*Z* = 3.039, *p* = 0.020) were tested, when compared to the untreated control. In the same context, Isonet^®^ L MISTERX843 at 4 units/ha performed better than Isonet^®^ L MISTERX843 at 2 units/ha (*Z* = −2.825, *p* = 0.038) (Figure 2c). Significant differences (*χ*^2^ = 19.731, *d.f.* = 4, *p* < 0.001) also emerged in the study site in Northern Italy, where a lower infestation rate was observed in plots managed with Isonet^®^ L MISTERX843 at 2 units/ha (*Z* = −3.012, *p* = 0.022) and 3 units/ha (*Z* = −3.464, *p* = 0.005) if compared to the standard, i.e., Checkmate^®^ Puffer^®^ LB at 3 units/ha (Figure 2c).

#### 3.2.2. Year 2018: Infested Flower Clusters and Bunches

**Figure 3:**
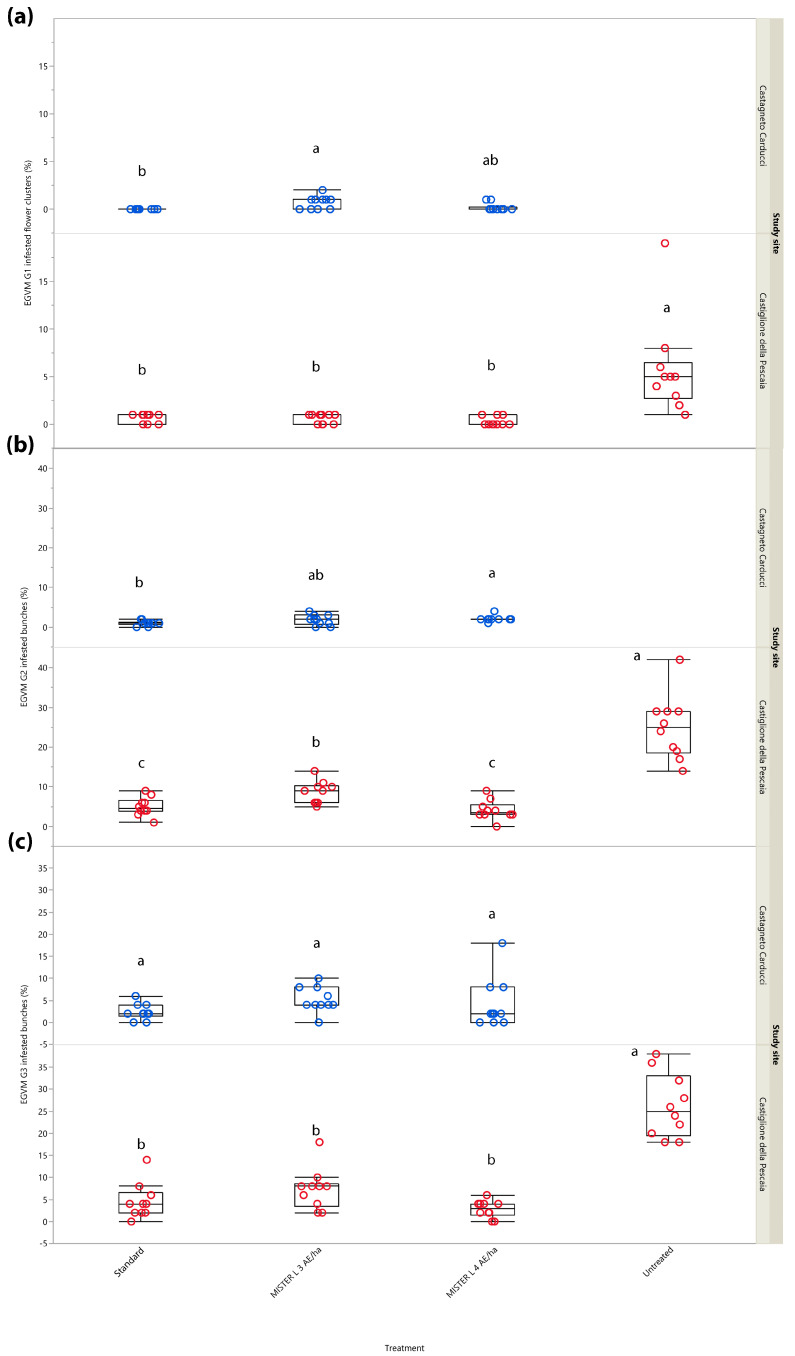
Impact of mating disruption using Isonet^®^ L MISTERX843 on the percentage of flower clusters (G1) or bunches (G2 and G3) infested by *Lobesia botrana* (EGVM) in Tuscany during 2018. Box plots indicate the median (solid line) within each box and the range of dispersion (lower and upper quartiles and outliers) of the infestation parameter. (**a**) G1: first generation; (**b**) G2: second generation; (**c**) G3: third generation (harvest). Within each generation and study site, different letters above box plots indicate significant differences between treatments (Kruskal–Wallis test followed by Steel–Dwass test, *p* < 0.05).

In 2018, MD experiments were conducted only in Central Italy (Tuscany) testing Isonet^®^ L MISTERX843 at 3 and 4 units/ha, which in 2017 were found to be more effective than 2 units/ha. G1 sampling showed significant differences in the percentage of infested flower clusters in Castagneto Carducci (*χ*^2^ = 9.396, *d.f.* = 2, *p* = 0.009), where the grower’s standard, i.e., Isonet^®^ L TT, showed a significant difference compared to plots managed with Isonet^®^ L MISTERX843 at 3 units/ha (*Z* = 2.781, *p* = 0.015). The latter was also noted in Castiglione della Pescaia (*χ*^2^ = 24.075, *d.f.* = 3, *p* < 0.001), where the percentage of infestation in the untreated control was significantly higher than in all MD treatments (Figure 3a).

At the end of the second generation (G2), the trial conducted in Castagneto Carducci showed the same significant differences (*χ*^2^ = 7.519, *d.f.* = 2, *p* = 0.023) noted in G1 (Figure 3b). The G1 trend was also confirmed for G2 in Castiglione della Pescaia (*χ*^2^ = 28.815, *d.f.* = 3, *p* < 0.001), with the addition of a significantly higher percentage of infested bunches in the plot managed with Isonet^®^ L MISTERX843 at 3 units/ha compared to the plot with Isonet^®^ L MISTERX843 at 4 units/ha (*Z* = −3.048, *p* = 0.012) and to the grower’s standard, i.e., Isonet^®^ L TT (*Z* = 2.676, *p* = 0.037) (Figure 3b).

At harvest (G3), no significant differences were found in Castagneto Carducci (*χ*^2^ = 5.022, *d.f.* = 2, *p* = 0.081) given the lack of an untreated control. Significant differences emerged in G1 were confirmed for G2 in Castiglione della Pescaia (*χ*^2^ = 25.974, *d.f.* = 3, *p* < 0.001) with a significantly higher percentage of infested bunches in the untreated control than in MD treatments (Figure 3c).

#### 3.2.3. Year 2017: Number of Nests

**Figure 4:**
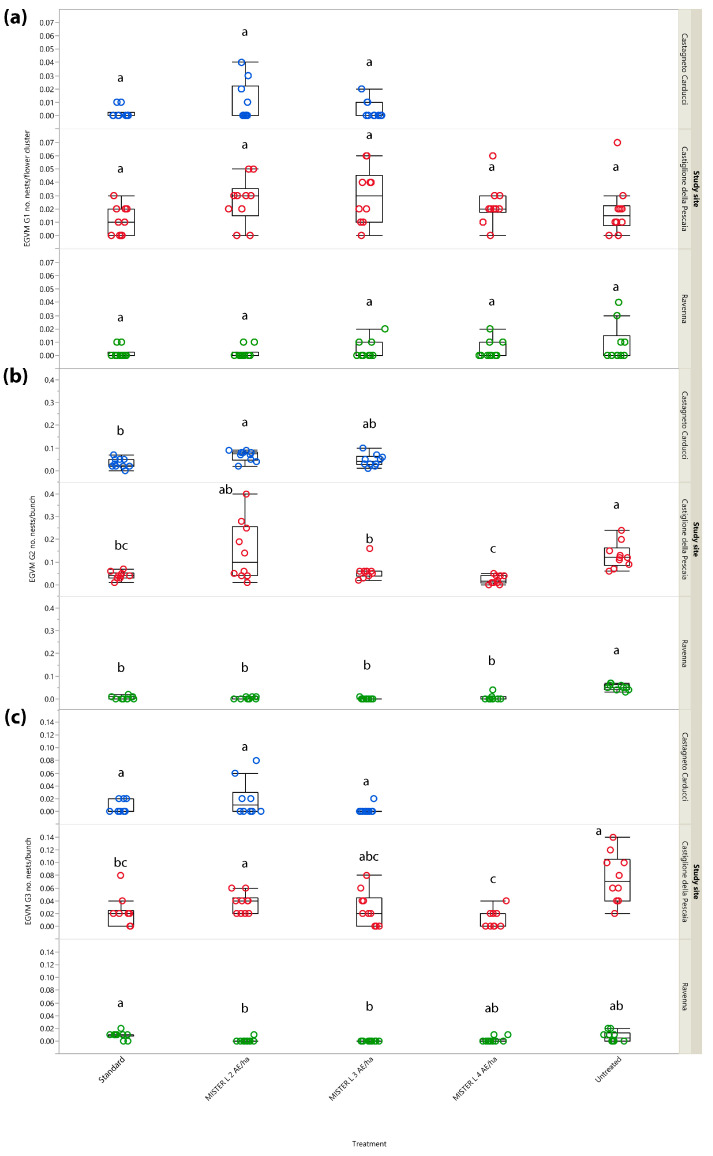
Impact of mating disruption using Isonet^®^ L MISTERX843 on the number of *Lobesia botrana* (EGVM) nests per flower cluster (G1) or bunch (G2 and G3) in Tuscany (Central Italy, in blue and red) and Emilia-Romagna (Northern Italy, in green) during 2017. Box plots indicate the median (solid line) within each box and the range of dispersion (lower and upper quartiles and outliers) of the infestation parameter. (**a**) G1: first generation; (**b**) G2: second generation; (**c**) G3: third generation (harvest). Within each generation and study site, different letters above box plots indicate significant differences between treatments (Kruskal–Wallis test followed by Steel–Dwass test, *p* < 0.05).

To provide a better assessment of MD performance on population density, the number of EGVM nests per flower cluster and bunch was also analyzed. In G1, no significant differences between the treatments were noted in Central and Northern Italy, i.e., Castagneto Carducci (*χ*^2^ = 1.639, *d.f.* = 2, *p* = 0.441), Castiglione della Pescaia (*χ*^2^ = 7.366, *d.f.* = 4, *p* = 0.118) and Ravenna (*χ*^2^ = 2.012, *d.f.* = 4, *p* = 0.734) (Figure 4a), most likely because, as for the percentage of infested inflorescences/clusters, it did not reach significance in G1 due to a very low pest pressure.

In G2, significant differences emerged in all study sites (i.e., Castiglione della Pescaia: *χ*^2^ = 25.365, *d.f.* = 4, *p* < 0.0001; Castagneto Carducci: *χ*^2^ = 8.121, *d.f.* = 2, *p* = 0.019; Ravenna: *χ*^2^ = 30.395, *d.f.* = 4, *p* < 0.0001), mainly reflecting results found in the assessment of infestation rates (Figure 4b).

In G3, significant differences emerged in Castiglione della Pescaia (*χ*^2^ = 21.655, *d.f.* = 4, *p* < 0.001) and Ravenna (*χ*^2^ = 19.019, *d.f.* = 4, *p* < 0.001), but not in Castagneto Carducci (*χ*^2^ = 4.253, *d.f.* = 2, *p* = 0.119) (Figure 4c) where both the test and reference MD dispensers showed a statistically comparable performance.

#### 3.2.4. Year 2018: Number of Nests

**Figure 5:**
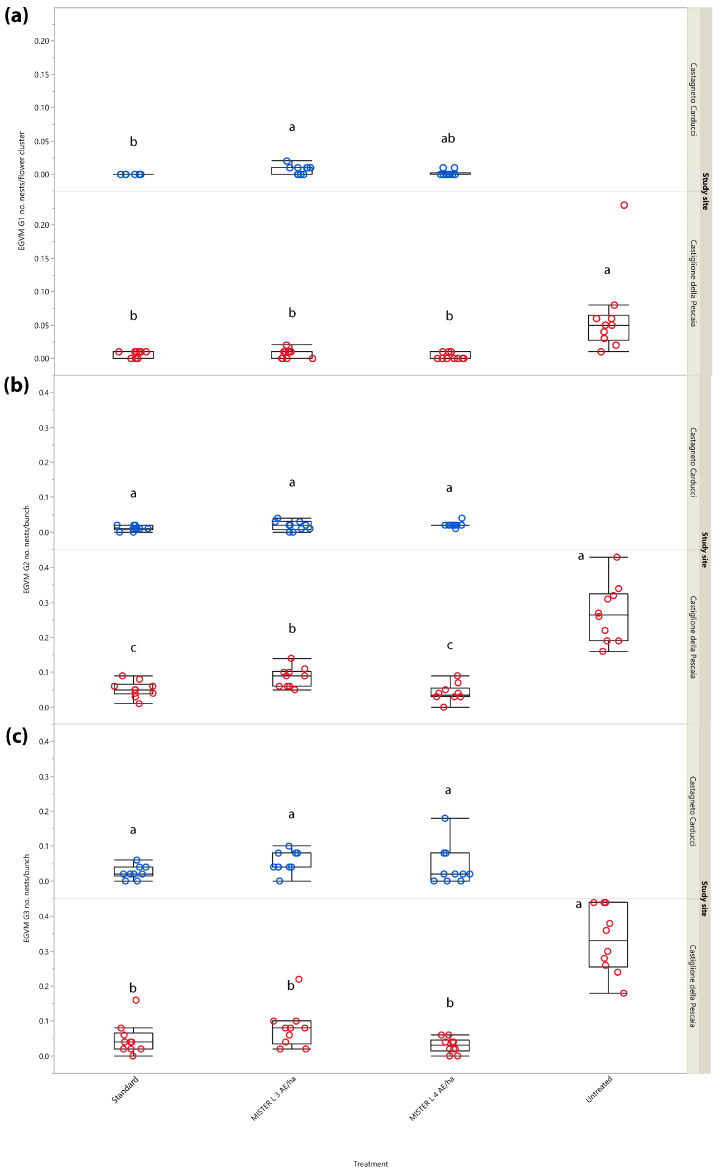
Impact of mating disruption using Isonet^®^ L MISTERX843 on the number of *Lobesia botrana* (EGVM) nests per flower cluster (G1) or bunch (G2 and G3) in Tuscany during 2018. Box plots indicate the median (solid line) within each box and the range of dispersion (lower and upper quartiles and outliers) of the infestation parameter. (**a**) G1: first generation; (**b**) G2: second generation; (**c**) G3: third generation (harvest). Within each generation and study site, different letters above box plots indicate significant differences between treatments (Kruskal–Wallis test followed by Steel–Dwass test, *p* < 0.05).

In G1, significant differences in the number of nests per flower cluster emerged in both Tuscan study sites (*χ*^2^ = 23.503, *d.f.* = 3, *p* < 0.001 and *χ*^2^ = 9.396, *d.f.* = 2, *p* = 0.009, Castiglione della Pescaia and Castagneto Carducci, respectively) (Figure 5a).

In G2, significant differences in the number of nests per bunch among the different treatments emerged in Castiglione della Pescaia (*χ*^2^ = 28.890, *d.f.* = 3, *p* < 0.001), but not in Castagneto Carducci (*χ*^2^ = 5.915, *d.f.* = 2, *p* = 0.052) (Figure 5b).

The G3 sampling carried out at harvest showed significant differences in terms of nests per bunch among the treatments in Castiglione della Pescaia (*χ*^2^ = 25.698, *d.f.* = 3, *p* < 0.001), but not in Castagneto Carducci (*χ*^2^ = 5.327, *d.f.* = 2, *p* = 0.069) (Figure 5c).

### 3.3. Economic Evaluation of Mating Disruption

In the present study, the MD economic evaluation showed that the whole cost per hectare was almost similar using active or passive release devices, since the higher cost of active release devices was balanced by a reduction in the manpower costs, due to the significant reduction in the time required to install and remove the devices (Table 4).

## 4. Discussion

Our results outline that the deployment of the MD experimental aerosol emitter Isonet^®^ L MISTERX843 provides a significant reduction in EGVM damage, if compared to the untreated control. Its performances are comparable, or even better, than those achieved testing passive (i.e., Isonet^®^ L TT) and active (i.e., Checkmate^®^ Puffer^®^ LB) MD devices currently marketed. Among the tested rates of the new aerosol emitter (respectively, 2, 3 and 4 dispensers/ha), a slight dose-response effect emerged, but this was not statistically confirmed throughout all the trials and assessments. Thus, the recommended application rate of the new MD product ranges from 2 to 4 dispensers/ha. To date, the few aerosol devices available for EGVM MD have been always evaluated in optimal wine-growing contexts characterized by large and fairly homogeneous vineyards [23,24]. For example, Lucchi et al. [3] managed EGVM populations through the application of MD aerosol emitters (2 units/ha) in Alfamén (Aragon region, Spain), an area characterized by a total surface area of 205,000 ha, of which 95,000 were covered by vines (Wine Regulatory Council, 2019). This viticultural context, consisting of vast and homogeneous vineyard areas, was particularly suitable for aerosol-based MD approaches [3]. On the other hand, the current study was carried out in areas large enough for MD (~3 ha), but in an agricultural context of small-sized vineyards with irregular orography (between the beginning and the end of the vineyards, the difference in height could vary up to 15 m with slopes of ~3%). Thus, these Italian vineyards were more patchy (with presence of wooded patches, other crops and water basins nearby) than the larger valleys of Aragon in Spain [27]. Of note, our results fully validated the efficacy of this novel aerosol emitter in the uneven environmental conditions typical of most Italian wine-growing areas. Broadly, these findings support the reliability of MD for managing EGVM as demonstrated by other authors [1,3,6,22,28,29,30,31], revealing the potential for innovation by developing efficient pheromone emitters to improve a strategy launched some time ago.

A substantial improvement in pest control strategies through the use of advanced aerosol emitters has been reported for several pests [32,33,34,35,36]. For instance, Burks and Thomson [32] investigated two aerosol emitter densities (2.5 and 5 units/ha) and different spraying frequencies for the management of *Amyelois transitella* (Walker) (Lepidoptera: Pyralidae) in almond and pistachio orchards; using aerosols for *A. transitella* MD improved cost-effectiveness compared to early MD systems. No significant differences emerged between the two dispenser densities per ha, corroborating our findings obtained by comparing three aerosol densities (2, 3 and 4 units/ha). On the other hand, the frequency of pheromone dispensing proved to be a key element for MD success. Indeed, the authors found a significant reduction in the number of males caught in the plot with a higher emission frequency compared with the lowest emission frequency [32]. Given the importance of this parameter, the dispensers we tested were sprayed from dusk to midnight, corresponding to the male flight activity period, as recently demonstrated by Lucchi et al. [26]. Moreover, the spray frequency varied according to the phenological phase of the vine (i.e., higher frequency during the sprouting phase of the vine to compensate for the higher dispersion of the pheromone due to low vegetation and lower frequency once the vine had produced a conspicuous vegetation wall) and the local temperature, to keep acceptable pheromone levels in the vineyard throughout the whole season. The choice of setting the spraying frequency as a function of vine phenology was based on several studies showing how the plant canopy determines the average concentrations of pheromones and their temporal and spatial distribution in MD trials [37,38]. Karg and Sauer [37] used a field electroantennogram (EAG) approach for measuring the dispersion, relative concentration of airborne pheromones, and their fluctuations in a defoliated vineyard during spring, as well as in a fully developed vineyard during the summer, both under MD. Synthetic pheromone concentrations were significantly lower in the defoliated vineyard than in the vineyard with well-developed vegetation, highlighting how the plant phenology is essential for MD performances. Similar results have been obtained in different agricultural contexts such as apple orchards [39], highlighting how a greater or lesser foliage presence on apple trees, due to seasonal effects or tree management, could positively or negatively influence MD performances. Thus, leaves can affect pheromone concentrations in two ways; by reducing wind speed due to physical resistance or by acting as an absorption and release reservoir due to their physico-chemical properties [39].

The tested active emitters allow for the optimization of synthetic pheromone distribution according to abiotic factors such as temperature (devices are equipped with thermal sensors regulating their activity exclusively once the thermal range agrees with that suitable for the target insect activity) as well as the phenology of the plant and increased efficiency, thus representing an important step in improving EGVM MD. Of note, upcoming developments could affect the emitted aerosol blend, reducing the pheromone component and coupling it with volatile organic compounds (VOCs), thus performing a synergic activity in attracting and confusing the males of a given target species [40,41]. For instance, Schmidt-Büsser et al. [40] showed that adding some plant volatiles (i.e., hexanol (99%), (*E*)-β-caryophyllene (99%), methyl salicylate (>99%), R(+)-limonene (>98%), ±linalool (racemic mixture, >97%), (*Z*)-3-hexen1-ol (>98%), (+)-terpinen-4-ol (99%), 1-octen-3-ol (racemic mixture, >97%), benzaldehyde (99%), (*E*)-2-hexenal (>95%) and 4,8-dimethyl-1(*E*),3,7-nonatriene (DMNT)) to the sex pheromone of *Eupoecilia ambiguella* (Hübner) (Lepidoptera: Tortricidae) meant that the ability of males to locate the source increased; this suggests that the simultaneous perception of host plant VOCs with the sex pheromone constituents helps *E. ambiguella* males to detect females on the host plants. Likewise, Yang et al. [41] found that adding some plant VOCs (i.e., codlemone, (*E*)-β-farnesene and (Z)-3-hexen-1-ol) to the *Cydia pomonella* L. (Lepidoptera: Tortricidae) pheromone increased its attractiveness to males of the target species. This knowledge could help in identifying new blends for more effective and cheaper MD programs, although further studies are required.

## 5. Conclusions

This study shows how newly developed sex pheromone aerosol emitters can help to optimize EGVM MD, being finely tunable for pheromone emission, and achieving comparable performance to hand-applied emitters, even in small-sized vineyards. However, to ensure the best performance of these active devices, several environmental and climatic aspects of the context, where the MD approach will be applied, should be evaluated. Indeed, as recently highlighted by Benelli et al. [24], there are several pros and cons to consider before their deployment. Vineyards with excessively irregular shapes do not allow for uniform MD towards the target pest, especially on the edges. Moreover, if the vineyard is in a context of strong dominant winds, the use of active emitters may not allow for the homogeneous distribution of the pheromone blend, which would be dispersed by the wind. Further research is still needed to improve this novel aerosol emitter and its emission rates, making it more adaptable to grapevine-growing contexts which are currently poorly suitable.

## Figures and Tables

**Table 1:** Location of the Italian study sites, pest history and year of *Lobesia botrana* mating disruption trials.

Trial	Site	Province	Region	Longitude	Latitude	Pest History (Infestation Level)	Year
1	Castiglione della Pescaia	Grosseto	Tuscany	10,937869 E	42,807762 N	Medium–high	2017
2	Castagneto Carducci	Livorno	Tuscany	10,622014 E	43,197295 N	Low	2017
3	Ravenna	Ravenna	Emilia-Romagna	12,28313333 E	44,3809472 N	Medium	2017
4	Castiglione della Pescaia	Grosseto	Tuscany	10,937869 E	42,807762 N	Medium–high	2018
5	Castagneto Carducci	Livorno	Tuscany	10,622014 E	43,197295 N	Low	2018

**Table 2:** Details of the Italian vineyards where *Lobesia botrana* mating disruption trials were performed.

Trial	Crop	Variety	Rootstock	Training System	Row Spacing (m)	Spacing within Rows (m)	Plant Age (Years)
1	Wine grape	Cabernet Sauvignon	3309	Low cordon	2	0.8	15–17
2	Wine grape	Cabernet Franc	SO4, 3309, RGM, 161-49C	Low cordon	2	0.8	13–18
3	Wine grape	Trebbiano	Kober 5BB	Guyot	3.4	1.4	10–11
4	Wine grape	Cabernet Sauvignon	3309	Low cordon	2	0.8	15–17
5	Wine grape	Cabernet Franc	SO4, 3309, RGM, 161-49C	Low cordon	2	0.8	13–18

**Table 3:** Treatments tested in the various study sites (ha = hectares).

Study Site and Year	Treatment 1	Treatment 2	Treatment 3	Grower’s Standard	Untreated Control
Castiglione della Pescaia, 2017	Isonet^®^ L MISTERX843 (2 units/ha)	Isonet^®^ L MISTERX843 (3 units/ha)	Isonet^®^ L MISTERX843 (4 units/ha)	Isonet^®^ L TT (250 units/ha)	Yes
Castagneto Carducci, 2017	Isonet^®^ L MISTERX843 (2 units/ha)	Isonet^®^ L MISTERX843 (3 units/ha)	-	Isonet^®^ L TT (250 units/ha)	No
Ravenna, 2017	Isonet^®^ L MISTERX843 (2 units/ha)	Isonet^®^ L MISTERX843 (3 units/ha)	Isonet^®^ L MISTERX843 (4 units/ha)	Checkmate^®^ Puffer^®^ LB (3 units/ha)	Yes
Castiglione della Pescaia, 2018	-	Isonet^®^ L MISTERX843 (3 units/ha)	Isonet^®^ L MISTERX843 (4 units/ha)	Isonet^®^ L TT (250 units/ha)	Yes
Castagneto Carducci, 2018	-	Isonet^®^ L MISTERX843 (3 units/ha)	Isonet^®^ L MISTERX843 (4 units/ha)	Isonet^®^ L TT (250 units/ha)	No

**Table 4:** Economic evaluation of the three products evaluated in this study for *Lobesia botrana* mating disruption.

Tested Product	Dose/ha (Tested)	Price/ha	Installation and Removal Time (Estimation)	Manpower Cost Installation and Removal ^§^	Total Cost/ha
Isonet^®^ L MISTERX843	3	180 EUR/ha **	40 min/ha/person	EUR 13	193 EUR/ha
Isonet^®^ L TT	250	121 EUR/ha **	2 h/ha/person	EUR 40	161 EUR/ha
Checkmate^®^ Puffer^®^ LB	3 *	210 EUR/ha **	40 min/ha/person	EUR 13	223 EUR/ha

** The density of application suggested by the producer is 2.5 units/ha. However, here we estimated the overall cost based on the density used in the trial. * Reported price/ha are suggested enduser price. **^§^** Cost of manpower: 20 EUR/h.

## Data Availability

The datasets generated during and/or analyzed during the current study are available from the corresponding author upon reasonable request.

## References

[1] Ioriatti C., Anfora G., Tasin M., De Cristofaro A., Witzgall P., Lucchi A. (2011). Chemical ecology and management of *Lobesia botrana* (Lepidoptera: Tortricidae). J. Econ. Entomol..

[2] Lucchi A., Bagnoli B., Cooper M.L., Ioriatti C., Varela L.G. (2014). The successful use of sex pheromones to monitor and disrupt mating of *Lobesia botrana* in California. IOBC/WPRS Bull..

[3] Lucchi A., Sambado P., Royo A.B.J., Bagnoli B., Conte G., Benelli G. (2018). Disrupting mating of *Lobesia botrana* using sex pheromone aerosol devices. Environ. Sci. Pollut. Res..

[4] Thiéry D., Louâpre P., Muneret L., Rusch A., Sentenac G., Vogelweith F., Iltis C., Moreau J. (2018). Biological protection against grape berry moths. A review. Agron. Sustain. Dev..

[5] Heit G., Sione W., Cortese P. (2015). Three years analysis of *Lobesia botrana* (Lepidoptera: Tortricidae) flight activity in a quarantined area. J. Crop Prot..

[6] Simmons G.S., Varela L., Daugherty M., Cooper M., Lance D., Mastro V., Carde R.T., Lucchi A., Ioriatti C., Bagnoli B. (2021). Area-Wide Eradication of the Invasive European Grapevine Moth *Lobesia botrana* in California, USA. Area-Wide Integrated Pest Management: Development and Field Application.

[7] Taret G.A.A., Azin G., Vanin M. (2021). Area-Wide Management of *Lobesia botrana* in Mendoza, Argentina. Area-Wide Integrated Pest Management.

[8] Cooper M., Varela L., Smith R., Whitmer D., Simmons G., Lucchi A., Broadway R., Steinhauer R. (2014). Growers, scientists and regulators collaborate on European grapevine moth program. Calif. Agric..

[9] Gilligan T.M., Epstein M.E., Passoa S.C., Powell J.A., Sage O.C., Brown J.W. (2011). Discovery of *Lobesia botrana* ([Denis & Schiffermüller]) in California: An invasive species new to North America (Lepidoptera: Tortricidae). Proc. Entomol. Soc. Washingt..

[10] Ioriatti C., Lucchi A., Varela L.G. (2012). Grape berry moths in western European vineyards and their recent movement into the New World. Arthropod Management in Vineyards.

[11] Benelli G., Lucchi A., Anfora G., Bagnoli B., Botton M., Campos-Herrera R., Carlos C., Daugherty M.P., Gemeno C., Harari A.R. (2023). European grapevine moth, *Lobesia botrana*: Part I, biology and ecology. Entomol. Gen..

[12] Hillocks R.J. (2012). Farming with fewer pesticides: EU pesticide review and resulting challenges for UK agriculture. Crop Prot..

[13] Civolani S., Boselli M., Butturini A., Chicca M., Fano E.A., Cassanelli S. (2014). Assessment of Insecticide Resistance of *Lobesia botrana* (Lepidoptera:Tortricidae) in Emilia-Romagna Region. J. Econ. Entomol..

[14] Hicks S.D., Wang M., Fry K., Doraiswamy V., Wohlford E.M. (2017). Neurodevelopmental delay diagnosis rates are increased in a region with aerial pesticide application. Front. Pediatr..

[15] Silver M.K., Shao J., Zhu B., Chen M., Xia Y., Kaciroti N., Lozoff B., Meeker J.D. (2017). Prenatal naled and chlorpyrifos exposure is associated with deficits in infant motor function in a cohort of Chinese infants. Environ. Int..

[16] Benelli G., Lucchi A., Anfora G., Bagnoli B., Botton M., Campos-Herrera R., Carlos C., Daugherty M.P., Gemeno C., Harari A.R. (2023). European grapevine moth, *Lobesia botrana*: Part II, prevention and management. Entomol. Gen..

[17] Godoy R., Aburto C., Lizana P., Venthur H., Palma-Millanao R., Méndez L., Panichini M., Moraga F., Bardehle L., Quiroz A. (2019). Antennal Morphology and Localization of a Pheromone-Binding Protein of *Lobesia botrana* (Denis & Schiffermüller) (Lepidoptera: Tortricidae). Neotrop. Entomol..

[18] Venthur H., Machuca J., Godoy R., Palma-Millanao R., Zhou J.J., Larama G., Bardehle L., Quiroz A., Ceballos R., Mutis A. (2019). Structural investigation of selective binding dynamics for the pheromone-binding protein 1 of the grapevine moth, *Lobesia botrana*. Arch. Insect Biochem. Physiol..

[19] Mori K., Tashiro T. (2004). Useful reactions in modern pheromone synthesis. Curr. Org. Synth..

[20] Altindisli F.O., Ozsemerci F. (2013). Efficacy evaluation of RAK 2 PRO dispensers against *Lobesia botrana* on Sultani Cekirdeksiz grapes in Turkey. IOBC WPRS Bull..

[21] Gavara A., Navarro-Llopis V., Primo J., Vacas S. (2022). Influence of weather conditions on *Lobesia botrana* (Lepidoptera: Tortricidae) mating disruption dispensers’ emission rates and efficacy. Crop Prot..

[22] Ricciardi R., Benelli G., Suma P., Cosci F., Di Giovanni F., Zeni V., Conte G., Marchesini E., Savino F., Ladurner E. (2022). One device for two pests: A new double dispenser for mating disruption of *Lobesia botrana* and *Planococcus ficus*. Entomol. Gen..

[23] Lucchi A., Ladurner E., Savino F., Cosci F., Benelli G. (2018). Eco-friendly pheromone dispensers—A green route to manage the European grapevine moth?. Environ. Sci. Pollut. Res..

[24] Benelli G., Lucchi A., Thomson D., Ioriatti C. (2019). Sex pheromone aerosol devices for mating disruption: Challenges for a brighter future. Insects.

[25] Gavara A., Vacas S., Navarro I., Primo J., Navarro-Llopis V. (2020). Airborne pheromone quantification in treated vineyards with different mating disruption dispensers against *Lobesia botrana*. Insects.

[26] Lucchi A., Sambado P., Juan Royo A.B., Bagnoli B., Benelli G. (2018). *Lobesia botrana* males mainly fly at dusk: Video camera-assisted pheromone traps and implications for mating disruption. J. Pest Sci. (2004).

[27] Albareda M., Guerra A., Monturiol F., Mateos P., Vicente G. (1961). Study of the Soils of the Ebro Valley.

[28] Baldessari M., Ioriatti C., Angeli G. (2013). Evaluation of Puffer ® CM, a release device of pheromone to control codling moth on apple in Italy. IOBC-WPRS Bull..

[29] Kast W.K. (2001). Twelve years of practical experience using mating disruption against *Eupoecilia ambiguella* and *Lobesia botrana* in vineyards of the Wuerttemberg region, Germany. Bulletin OILB/SROP.

[30] Varner M., Lucin R., Mattedi L., Forno F. (2001). Experience with mating disruption technique to control grape berry moth, *Lobesia botrana*, in Trentino. IOBC/WPRS Bull..

[31] Vassiliou V.A. (2009). Control of *Lobesia botrana* (Lepidoptera: Tortricidae) in vineyards in Cyprus using the mating disruption technique. Crop Prot..

[32] Burks C.S., Thomson D.R. (2019). Optimizing Efficiency of Aerosol Mating Disruption for Navel Orangeworm (Lepidoptera: Pyralidae). J. Econ. Entomol..

[33] Higbee B.S., Burks C.S., Cardé R.T. (2017). Mating Disruption of the Navel Orangeworm (Lepidoptera: Pyralidae) Using Widely Spaced, Aerosol Dispensers: Is the Pheromone Blend the Most Efficacious Disruptant?. J. Econ. Entomol..

[34] Mcghee P.S., Miller J.R., Thomson D.R., Gut L.J. (2016). Optimizing Aerosol Dispensers for Mating Disruption of Codling Moth, *Cydia pomonella* L.. J. Chem. Ecol..

[35] Mori B.A., Evenden M.L. (2015). Challenges of Mating Disruption Using Aerosol-Emitting Pheromone Puffers in Red Clover Seed Production Fields to Control *Coleophora deauratella* (Lepidoptera:Coleophoridae). Environ. Entomol..

[36] Ortiz A., Marti J., Tudela A., Rodr Á., Sambado P. (2021). Mating disruption of the olive moth *Prays oleae* (Bernard) in olive groves using aerosol dispensers. Insects.

[37] Karg G., Sauer A.E. (1997). Seasonal variation of pheromone concentration in mating disruption trials against European grapevine moth *Lobesia botrana* measured with elctroantennograms. J. Chem. Ecol..

[38] Sauer A.E., Karg G. (1998). Variables affecting pheromone concentration in vineyards treated for mating disruption of grape vine moth *Lobesia batrana*. J. Chem. Ecol..

[39] Karg G., Suckling D.M., Bradley S.J. (1994). Absorption and release of pheromone of *Epiphyas postvittana* (Lepidoptera: Tortricidae) by apple leaves. J. Chem. Ecol..

[40] Schmidt-Büsser D., von Arx M., Guerin P.M. (2009). Host plant volatiles serve to increase the response of male European grape berry moths, *Eupoecilia ambiguella*, to their sex pheromone. J. Comp. Physiol..

[41] Yang Z., Bengtsson M., Witzgall P. (2004). Host plant volatiles synergize response to sex pheromone in codling moth, *Cydia pomonella*. J. Chem. Ecol..

